# Assessment of proportional hazard assumption in aggregate data: a systematic review on statistical methodology in clinical trials using time-to-event endpoint

**DOI:** 10.1038/s41416-018-0302-8

**Published:** 2018-11-13

**Authors:** Eliana Rulli, Francesca Ghilotti, Elena Biagioli, Luca Porcu, Mirko Marabese, Maurizio D’Incalci, Rino Bellocco, Valter Torri

**Affiliations:** 1 Laboratory of Methodology for Clinical Research, Oncology Department, Istituto di Ricerche Farmacologiche Mario Negri IRCCS, Milan, Italy; 2 Department of Statistics and Quantitative Methods, University of Milano-Bicocca, Milano, Italy; 3 Laboratory of Molecular Pharmacology, Oncology Department, Istituto di Ricerche Farmacologiche Mario Negri IRCCS, Milan, Italy; 4 Laboratory of Cancer Pharmacology, Oncology Department, Istituto di Ricerche Farmacologiche Mario Negri IRCCS, Milan, Italy; 5 Department of Medical Epidemiology and Biostatistics, Karolinska Institutet, Stockholm, Sweden

**Keywords:** Non-small-cell lung cancer, Randomized controlled trials

## Abstract

**Background:**

The evaluation of the proportional hazards (PH) assumption in survival analysis is an important issue when Hazard Ratio (HR) is chosen as summary measure. The aim is to assess the appropriateness of statistical methods based on the PH assumption in oncological trials.

**Methods:**

We selected 58 randomised controlled trials comparing at least two pharmacological treatments with a time-to-event as primary endpoint in advanced non-small-cell lung cancer. Data from Kaplan–Meier curves were used to calculate the relative hazard at each time point and the Restricted Mean Survival Time (RMST). The PH assumption was assessed with a fixed-effect meta-regression.

**Results:**

In 19% of the trials, there was evidence of non-PH. Comparison of treatments with different mechanisms of action was associated (*P* = 0.006) with violation of the PH assumption. In all the superiority trials where non-PH was detected, the conclusions using the RMST corresponded to that based on the Cox model, although the magnitude of the effect given by the HR was systematically greater than the one from the RMST ratio.

**Conclusion:**

As drugs with new mechanisms of action are being increasingly employed, particular attention should be paid on the statistical methods used to compare different types of agents.

## Introduction

In many clinical and observational studies, especially in oncology, the quantity of main interest is the length of time before an event occurs. In oncology, especially in phase III trials, the outcomes of interest are death, progression or relapse of the disease. In this setting, a time-to-event endpoint is used and survival analysis is performed to analyse the data.

Different methods can be used for survival analysis. Among the non-parametric methods, Kaplan–Meier (KM) estimator is the most common.^1^ The Log-rank test can be used to evaluate whether KM curves are statistically different. The Cox proportional hazards (PH) model^2^ is the most common approach^3–5^ to detect and estimate the effect of several risk factors on survival. The measure of association estimated by the Cox PH model is the hazard ratio (HR), which is, with two treatment groups, the ratio of the hazard of the outcome of interest in the treated to the control group. The hazard rate represents the instantaneous risk of the event of interest occurrence. The Cox model does not require any parametric assumptions about the shape of the baseline hazard function, but relies on the proportionality of the hazards, so the HR is assumed constant over time. When the PH assumption fails, the HR estimated by the Cox model depends on follow-up time^6,7^ and it has also been seen that, under these circumstances, the Log-rank test has a lower power.^8^

When the PH assumption is not met, the effectiveness estimates are likely to be not representative for the whole intervention period and the effect of this bias, in case of meta-analyses, will be carry over to the analyses of aggregate data. Although survival curves convergence and crossings are common in medical research,^9,10^ too little attention is paid to this issue and the statistical test of PH assumption is rarely reported in clinical trial publications. It has been reported that crossing survival curves are common when one of the treatment compared offers a short-term benefit, but no long-term advantages^8^ or when the treatments being compared have different biological mechanisms of action, or differently responsive sub-populations are included.^11^ When individual patient data are available, there are several options for assessing the PH assumption.^12,13^ However, no ready to use methods exist to test the validity of PH assumption when only aggregate data are available. Testing the PH assumption on aggregate data is therefore needed to guarantee the validity of meta-analysis results.

Several methods exist to analyse time-to-event endpoints when the HR is not an adequate summary statistic for treatment effect.^14,15^ The Restricted Mean Survival Time (RMST) is an alternative to the HR.^11,14^ The RMST does not need any model assumptions, such as the hazards to be proportional, and is readily interpretable as ‘life expectancy’ between the time of randomisation and a relevant time point.

We conducted a systematic review of randomised phase II and III clinical trials comparing different types of pharmacological treatment in patients with advanced non-small cell lung cancer (NSCLC) with the aim of assessing the appropriateness of survival analysis based on the PH assumption. A method to test the PH assumption on aggregate data was proposed and factors influencing it were also investigated.

In the absence of PH, the Cox model results were compared to those based on the RMST to investigate the robustness of the conclusions drawn by looking at the biased average HR estimate.

The choice of focusing our analysis on the oncological area was driven by the publication in the last years of some important trials where the PH assumption clearly failed (e.g. IPASS^16^ and ICON7^17^).

We focused on NSCLC since this is the most common cause of cancer deaths worldwide^18^ and although recent preclinical studies have improved the knowledge of the molecular mechanism governing the cancer cell, the majority of oncologic patients still do not benefit from new clinical therapy. NSCLC therefore represents a research area where the development of drugs with new mechanisms of action is very active.

## Methods

### Eligibility criteria

All phase II and III randomised controlled trials (RCTs) published from January 2004 to January 2015 comparing two or more systemic therapies in patients with advanced NSCLC were considered. Studies using a time-to-event primary endpoint, such as overall survival (OS), progression-free survival (PFS) and time-to-progression (TTP) were eligible. We excluded studies assessing different strategies for the same agent (i.e. different schedules or doses), using placebo, radiotherapy or surgery alone as comparator. Studies terminated early were also excluded.

### Search strategy

We searched Medline and Embase databases using search terms including non-small cell lung cancer, randomised clinical trials, chemotherapy, vascular endothelial growth factor receptor inhibitor, tyrosine-kinase inhibitor and a list of approved drugs for NSCLC. The complete search strategy is available in Supplementary Table S1. Two reviewers independently evaluated both the titles and the abstracts to ensure eligibility. Full manuscripts of potentially eligible trials were read to identify studies to be included. A third reviewer solved disagreements.

### Data extraction

Two reviewers, using a data extraction form, independently recorded the study design, patient and treatment characteristics, methodological and statistical features. As above, a third reviewer, who referred back to the original article, solved any differences in data extraction.

### Treatments and comparisons

According to their mechanism of action, treatments were classified in four categories:^1^ conventional therapy (drugs causing DNA damage or inhibition of DNA synthesis),^2^ biologics (antibodies against growth and angiogenic factors),^3^ tyrosine-kinase inhibitor (TKI) and^4^ miscellaneous group. The latter includes inhibitors of kinase other than tyrosine-kinase (i.e. protein kinase alpha and cand PI-3 kinase), metalloproteinase inhibitors, inhibitors of proapoptotic proteins and Toll-like receptor-9 agonist.

Studies were classified as those comparing treatments belonging to different categories or those comparing treatments within the same category.

### Statistical methods

Only papers reporting KM curves with patients at risk at more than two time points and separately for each treatment group were included in the statistical analysis; the papers not fulfilling this condition were only described. For each study analysed, the number of event-free patients at each time point and the respective survival probabilities were extracted from the published KM curves. Then, during each interval, we estimated the number of patients at risk, the number of censored patients and the number of events. These items were used to estimate the ln(HR) and its variance according to the methodology proposed by Williamson.^19^ The PH assumption was tested graphically using a plot of the log cumulative hazard, where the logarithm of time is plotted against the estimated log cumulative hazard calculated as ln[-ln (S(t))].^20^ If the curves for the two treatment groups were approximately parallel, the PH assumption was deemed reasonable. The graphical method is useful for visualising clear departures from the PH assumption, but due to the arbitrary assessment of these plots, a formal test was then applied. For each study, the relationship between the ln(HR) estimate at each time point and the follow-up time was described by a forest plot. To formally assess PH assumption a fixed-effect meta-regression within each study was conducted. The ln(HR) at each time point was the outcome of interest and the follow-up time was included in the model as the only covariate. It has been previously showed that, within each study, the HR(t) for *t* = 1,…,*p* are independent^21^ and therefore log(HR) estimates at different time points were treated as being obtained from different studies, as it is the case in the classic setting of meta-regression. If the ln(HR) was found to change over the follow-up time, as a result of a statistically significant estimate in meta-regression, the PH hypothesis was rejected. For the articles with co-primary endpoints, only one of them was included in the analysis. The endpoint with a statistically significant result was chosen; if none reported a significant result, the one with most patients at risk at the beginning of the follow-up was considered. For articles with more than two treatment arms, the PH assumption was tested separately for each comparison. The RMST was obtained for each study calculating the area under the KM curve for each interval using the method of trapezoids. The RMST was calculated up to last available follow-up time, defined as the greatest time point with still patients at risk reported under the KM curve in each arm. The follow-up time chosen was the same for all the treatment arms within study, unless one of the two treatment arms being compared reaches a value of zero in the survival curve. In this case the RMST of the other arm was calculated beyond the time point in which the first curve reaches the 0, until its last available time point. The RMST variance was calculated according to Klein et al.^22^ Differences in RMST between arms, the ratio and the relative Z test to assess the statistical significance of the difference were computed. Fisher’s exact test was used to investigate the association between absence of PH and study characteristics such as treatment comparisons (different categories vs. same category), endpoint (OS vs. other) and study results (significant results vs. non-significant). The two-sided significance level was set at 0.05 to test associations with study characteristics and to test RMST difference, and at 0.10 for meta-regression analysis. We used the web-based tool WebPlotDigitizer available online at https://automeris.io/WebPlotDigitizer/ to extract data from the KM curves.

Meta-regression was conducted in Stata (version 13) with the *vwls* variance-weighted least squares command.^23^

## RESULTS

The databases search identified 1078 records. Of these, 882 were excluded on the basis of the abstract evaluation, and the full text was obtained for the remaining 196. One additional paper was identified from the references of selected articles and added to the list of articles to be screened because potentially eligible. Full-text review led to the exclusion of 82 studies, not meeting the inclusion criteria: 32 did not have a time-to-event endpoint, 22 had a different aim, in 11 the control group received placebo or no therapy, for 8 the trial was terminated prematurely (3 trials were terminated due to low recruitment, 2 were stopped for futility, 2 for a high rate of unexpected mortality and toxicity, 1 for new evidences external from the trial), 2 were study protocols, 5 were updates of other articles included with no additional information, for 2 the full text was not available. Since 52 of the 115 articles we selected for data extraction did not report the number of patients at risk and 5 gave only two time points, survival data were extracted from the remaining 58 articles (Supplementary Figure S1).^16,24–80^ Four of these had co-primary endpoints^47,62,69,76^ and four had three treatment arms.^26,64,77,81^ Details of the 58 trials regarding settings, interventions and methodological characteristics are shown in Supplementary Tables S2 and S3.

### Study characteristics

Table 1 summarises the 115 studies in the review, according to their inclusion/exclusion from the statistical analysis. For 56 (49%) studies, OS was the primary endpoint, while for 52 (45%) PFS was used. The median number of randomised patients was 332. It is worth noting that 12 (10%) trials randomised fewer than 100 participants. Out of 115 articles, 102 (89%) reported the sample size. Among these, only 70 (69%) articles reported the number of events needed to reach the desired statistical power. The remaining 32 (31%) articles only reported the total number of patients to be included. Of the 70 (61%) articles reporting the number of events required, only 49 (70%) described the number of events reached. In particular 40 (82%) of these reported a number of events observed greater than 95% of those planned, while 9 (18%) did not reach the 95% of events required. Only four (3%) out of 115 studies reported that the PH assumption was tested and no evidence of failure of PH was seen in any of these studies. In the NVALT-10 study^82^ the PH assumption was assessed from scaled Schoenfeld residuals. In the TAILOR study^32^ the PH assumption was verified using graphic plots of Schoenfeld residuals over time, and by adding time-dependent variables in the model to test their statistical significance. In the FASTACT-2 trial^79^ the PH assumption was assessed graphically by plotting log–log survival functions for the two treatment groups. In the last trial^83^ the PH assumption was informally assessed by simply looking at the survival functions. In a further study^41^ the absence of PH was mentioned but the method used to test it was not described. From 115 studies, 128 comparisons were obtained. According to our classification, 53 (41%) involved treatments with the same mechanism of action while 75 (59%) compared treatments with different mechanisms (Table 2).

**Table 1:** Characteristics of the studies included in the review

	Excluded from statistical analysis (*N* = 57)		Included in statistical analysis (*N* = 58)		Total (*N* = 115)	
	*N*	%	*N*	%	*N*	%
Phase						
I–II	1	2	0	0	1	1
II	19	33	19	33	38	33
II–III	1	2	1	2	2	2
III	36	63	38	65	74	64
Type of study—centre						
Multicentre	43	75	57	98	100	87
Single centre	14	25	1	2	15	13
Blinding						
Yes	10	18	22	38	32	28
No	47	82	36	62	83	72
Primary Endpoint						
OS	32	56	24	41	56	49
PFS	20	35	32	55	52	45
TTF	1	2	0	0	1	1
TTP	4	7	2	4	6	5
Proportionality assessed						
Yes	2^a^	4	2	3	4	3
No	55	96	56	97	111	97
Sample size calculation						
Number of events reported	25	44	45	78	70	61
Only number of patients reported	23	40	9	15	32	28
Not provided	9	16	4	7	13	11
Reached >95% of target events						
Yes	14	82	26	81	40	82
No	3	18	6	19	9	18
Number of patients analysed						
Median	302		379		332	
IQR	154–440		175–772		168–595	
Minimum–Maximum	48–1725		60–1433		48–1725	

^a^In one study the proportionality assumption was informally assessed by looking at the survival functions

*N* number, *OS* overall survival, *PFS* progression-free survival, *TTF* time to failure, *TTP* time to progression, *IQR* interquartile range

**Table 2:** Treatment comparisons investigated by the articles included in the review

	Excluded from statistical analysis		Included in statistical analysis		Total	
	*N*	%	*N*	%	*N*	%
Same treatment comparison	33	50	20	32	53	41
1 vs 1	30	91	15	75	45	85
3 vs 3	3	9	5	25	8	15
Different treatments comparison	33	50	42	68	75	59
1 vs 3	5	15	13	31	18	24
1 vs 1 + 2	6	18	9	21	15	20
1 vs 1 + 3	12	37	14	34	26	35
1 vs 1 + 4	6	18	0	0	6	8
1 vs 1 + 2 + 3	1	3	0	0	1	1
2 vs 1 + 2	0	0	2	5	2	3
2 vs 2 + 3	0	0	1	2	1	1
3 vs 1 + 3	2	6	0	0	2	3
3 vs 2 + 3	0	0	3	7	3	4
3 vs 4	1	3	0	0	1	1
Total	66	100	62	100	128	100

*1* conventional therapy (drugs causing DNA damage or inhibition of DNA synthesis), *2* biologics (antibodies against growth and angiogenic factors), *3* tyrosine-kinase inhibitor (TKI), *4* miscellaneous group

### PH assumption assessment

The median number of time points with the corresponding number of patients at risk reported under the KM curves was six (Interquartile range (IQR) 4–9). The median decrease in the number of patients at risk from the start of the observation period to the first time point reported was 34% (IQR 18–49%). According to our meta-regression analysis, in 12 (19%) out of 62 treatment comparisons the PH assumption was violated.^16,25,26,38,41,49,52,66,71,72,75,78^

**Fig. 1:**
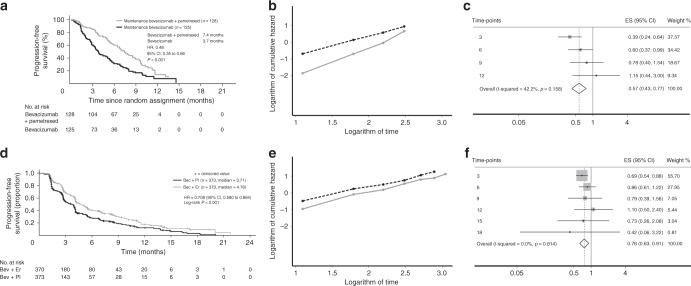
**a**–**c** Example in which proportional hazard assumption is violated:^25^ **a** Published KM curves; **b** Log–log plot; **c** Forest plot. **d**–**f** example in which proportional hazard assumption is verified:^42^ **d** Published KM curves; **e** Log–log plot; **f** Forest plot

Supplementary Figures S2, S3 and S4 report all the log–log plots and the forest plots of these 12 articles. For illustrative purposes Fig. 1 reports two studies, in the first the PH assumption was violated,^25^ while in the second the PH assumption was not rejected.^42^

Comparisons of treatments with a different mechanisms of action were significantly associated (*P* = 0.006) with violation of PH assumption (Table 3). Ten (83%) studies in which the assumption was violated and 27 (54%) in which the PH was met, had PFS as primary endpoint, but this difference did not reach statistical significance (*P* = 0.101). Among the nine superiority trials in which absence of PH was detected, seven (78%) gave statistically significant results (*P* = 0.069). Among the seven non-inferiority trials, four (57%) satisfied the PH assumption. In the latter, the experimental treatment was non-inferior to the control arm. In the other three (43%), the PH assumption was rejected and the experimental arm could not be considered non-inferior to the control (*P* = 0.029).

**Table 3:** Association between proportional hazard assumption results and study characteristics

	PH assumption violated		*P* Fisher’s exact test
	No	Yes	
Treatments			
Same treatment comparison	20 (40%)	0 (0%)	
Different treatments comparison	30 (60%)	12 (100%)	.006
Primary endpoint			
OS	23 (46%)	2 (17%)	
PFS/TTP/TTF	27 (54%)	10 (83%)	.101
Superiority trial			
Positive result (superiority demostrated)	19 (41%)	7 (78%)	
Negative result (superiority not demostrated)	27 (59%)	2 (22%)	.069
Non-inferiority trial			
Positive result (non-inferiority demonstrated)	4 (100%)	0 (0%)	
Negative result (non-inferiority not demonstrated)	0 (0%)	3 (100%)	.029

*PH* proportional hazard, *OS* overall survival, *PFS* progression-free survival, *TTP* time to progression, *TTF* time to failure

### Restricted mean survival time

HR, RMST (difference and ratio) and the relative statistical tests for the nine superiority trials in which the PH assumption was rejected, are reported in Table 4. In all these studies, results with the RMST difference corresponded to the conclusions drawn by the authors, based on Cox models as far as the statistical significance is concerned. Though the HR and the RMST ratio have different meanings in quantifying differences between arms, the two measures of the magnitude of treatment effect are on the same relative scale and can be easily compared. When the HR is plotted against the RMST ratio (Figure S5), it can be observed that the magnitude of the treatment effect given by the HR is systematically greater than the RMST ratio. The tendency does not seem related to the PH assumption violation as shown in the two regression lines in Figure S5.

**Table 4:** Comparison of the RMST results and the HR results in studies with PH assumption violated

RMST results						HR results	
Study	Control arm (months)	Experimental arm (months)	Difference (months)	*P* test Z	Ratio^a^ 95%CI	HR	*P*
Belani^26^	9.51	10.15	0.65	0.730	0.94 (0.65–1.36)	0.89	0.360
Reck^66^	3.42	4.18	0.76	0.018	0.82 (0.69–0.97)	0.79	0.002
Lee^49^	3.70	4.92	1.23	0.150	0.76 (0.51–1.10)	0.73	0.040^b^
Janne^41^	8.82	9.65	0.82	0.610	0.91(0.65–1.30)	0.80	0.210^b^
Barlesi^25^	4.88	7.24	2.37	<0.001	0.67 (0.53–0.86)	0.48	<0.001
Shaw^72^	5.95	9.27	3.33	0.004	0.64 (0.47–0.88)	0.49	<0.001
Seto^71^	11.35	16.48	5.13	<0.001	0.69 (0.55–0.87)	0.54	0.002
Solomon^75^	8.00	14.13	6.13	<0.001	0.57 (0.45–0.72)	0.45	<0.001
Wu^78^	5.63	12.29	6.66	<0.001	0.46 (0.37–0.57)	0.28	<0.001

*RMST* restricted mean survival times, *HR* hazard ratio, *KM* Kaplan–Meier

^a^Calculated as ratio between RMST in the control and RMST in the experimental arm

^b^One-sided, 95%CI, 95% confidence intervals

## DISCUSSION

An important finding that emerged from this analysis was the significant association between the kind of treatments compared and the absence of PH. New oncological treatments were frequently compared to a conventional therapy with a different mechanism of action. This is frequently reflected in a different course of the disease progression and might explain why, when treatments with different mechanisms are compared, the hazards are not proportional. As drugs with different mechanisms of action are increasingly investigated, due attention must be paid to the statistical methods used in these circumstances.

From our review stands out that only 3% of the studies analysed reported or mentioned the PH test. In these studies, the conclusion drawn by authors were concordant with the result from meta-regression testing. In one study,^41^ where it was found that hazards were nonproportional, they still decided to report and interpret HR without discussing possible implications. The proposed method showed that in 12 out of 56 treatment comparisons (19%) the PH assumption was not satisfied. This means that the statistical methods applied to analyse the treatment effect might be not adequate. When the PH assumption fails, the RMST difference can be used as a primary endpoint as it does not require hazards to be proportional. Even when the PH assumption appears to be satisfied, RMST may be a useful secondary measure because it gives a different, but complementary information.

We acknowledge that RMST also has some limitations. Since it depends on the time point chosen to calculate it, an inappropriate choice may give misleading results. A further limit, in the study design setting, concerns the within-group variances hypothesis for sample size calculation. However, when comparing conclusions based on the Cox model to those based on the RMST difference no discrepancy was observed.

Trinquart et al.^84^ analysed 54 RCTs, reconstructing individual patient data and calculating both the HR and the RMST ratio for each trial. The results obtained with these two measures were consistent and this behaviour was independent of the presence or absence of PH. Despite the agreement between RMST difference and HR on the statistical significance of the treatment effect, they provided empirical evidence that the treatment effect measure based on RMST yielded more conservative estimates than the ones based on HR. We found the same behaviour, with HR systematically overestimating the treatment effect compared to the RMST ratio.

In our review, the comparisons of treatments with different mechanisms of action involved conventional therapies compared with TKI or biological therapies. In the last few years, immunotherapy has emerged as a promising therapeutic strategy and has radically transformed the therapeutic landscape for NSCLC.^85,86^ In this setting too the problem of non-PH can be expected since immunotherapy efficacy translates into long-term survival and delayed clinical effects.^87^ When conventional therapy is compared with immunotherapy, in case of non-PH an underestimation of treatment effect can be expected when HR is used to measure it. When the PH assumption is unmet, based on our findings the Cox model still seems to bring to the right conclusions. It is important to note, however, that the estimate obtained becomes time-dependent and might not appropriately describe the phenomenon investigated.

Here we propose a method for testing the PH assumption when only aggregate data are available. The suggested method relies on published KM curves and, after data have been extracted, meta-regression is used to assess the PH assumption by testing for a linear trend of HRs with time. Systematic reviews and meta-analyses of well-designed and executed RCTs have the potential to provide the highest levels of evidence to support diagnostic and therapeutic decisions. To guarantee the accuracy of the meta-analysis results, however, it is important to assess the PH assumption for each considered trial and to evaluate the impact of the inclusion of trials not satisfying the assumption on the meta-analysis results. Even if the authors of the original study have not checked whether the PH assumption is satisfied or not, the method proposed here can be applied. Since a pooled analysis of studies with non-PH can produce an over- or underestimation of the efficacy comparison, this tool could be very useful when the aim is to conduct a meta-analysis. In case of non-PH detection in one or more than one study to be included in the meta-analysis different approaches might be adopted: e.g. one possibility is to include all the studies in the meta-analysis and run sensitivity analyses excluding non-PH studies; otherwise one can use alternative summary measures beyond HR which do not require the proportionality of the hazards such as survival at time *t* or RMST.

The main limit of the method is that it depends on the quality of published KM curves. Moreover, to estimate the number of events and censored in each interval an assumption must be made about the censoring mechanism. Censoring is assumed to be constant within each interval. The smaller the interval, the more likely the assumption will be met. Once again, the size of the interval depends on the number of time points reported under the KM curve. If these are relatively few, an appropriate assessment of the PH assumption is not possible, leading to low statistical power of the test. It is worth investigating how many time points are needed to draw appropriate conclusions with regard to the PH assumption. Future developments include comparisons of the conclusions obtained with individual patient data using the consolidated methods and those from aggregate data using meta-regression.

Guyot et al.^88^ recently proposed a method that allows simulating (or approximating) patient-level data based on KM curves. The advantage of using simulated patient-level data is that once simulated patient-level data are obtained, standard methods to assess the PH assumption can be used. This probably leads to a better chance of detecting the departure from the PH assumption, if any, compared to our method which could be less sensitive. Even if our method and that proposed by Guyot were presented as tools for testing PH assessment from published data, the aim and the context of application can be different. The method proposed by Guyot is very helpful and necessary when the aim of the meta-analysis is to pool survival data between studies or to analyse data with different survival models than the ones used in the published papers. The performance of this method relies on the information reported in the original papers. The authors stated that if the total number of events and the numbers at risk other than at time zero are not provided, the algorithm may produce poor results. Furthermore, it is a time-consuming process: it takes about half an hour to obtain the initial input data for one KM curve (i.e. one hour for each study in case of a two arm trial). Our aim was to propose a simpler method to test PH assumption from aggregate data when performing a meta-analysis. In this setting, it is very important to have an easy and quick to use tool. The meta-regression can be considered a valid alternative to the method proposed by Guyot because it does not require paid software and the time required to complete the process is less. However, a formal comparison between the two methods would be useful to investigate their properties in different settings.

A further limit of our work is that we applied our method in only 45% of eligible papers. This was because of the missing information on patients at risk at different time points. Parmar et al.^21^ proposed methods to estimate not only the number of censored and the number of events in each interval, but also the number of patients at risk. These methods, however, require further assumptions resulting in estimates even more approximated. For this reason, we decided to minimise the number of assumptions to be made, including only papers with all information available.

The conclusions from the meta-regression were compared to the results from the consolidated graphic method in which the logarithm of the time is plotted against the estimated log cumulative hazard. This was done in order to analyse the appropriateness of conclusions drawn according to meta-regression results. When the sample size is small, this method may lack power to detect deviations from PH; while for large sample sizes, hypothesis tests may be over sensitive to slight deviations from this assumption. In our sample, the results obtained with the meta-regression were in line with the log–log plots in all the studies. Despite the subjectivity of the graphic method, in all the 12 studies in which the PH assumption was rejected the graph agrees with the test results, indeed the curves were clearly not parallel. Moreover, for the four trials in which the authors tested the PH assumption, the conclusions with meta-regression were the same as the ones reported in the original papers.

Although further investigations are needed, the results we observed do suggest that meta-regression is a valid method for testing the PH assumption when only aggregate data are available.

## Electronic supplementary material

Supplemetary material

supplementary figure S1

supplementary figure S2

supplementary figure S3

supplementary figure S4

supplementary figure S5

permission to pubblish fig 1d

permission to pubblish fig 1a
