# Supplementary material for: Assessment of proportional hazard assumption in aggregate data: a systematic review on statistical methodology in clinical trials using time-to-event endpoint
Source: Br J Cancer. 2018 Nov 13;119(12):1456–63. doi: 10.1038/s41416-018-0302-8 (PMC6288087; doi:10.1038/s41416-018-0302-8)
Supplement: Supplementary file 1 — Supplemetary material [file 41416_2018_302_MOESM1_ESM.docx]

**Assessment of proportional hazard assumption in aggregate data: a systematic review on statistical methodology in clinical trials using time-to-event endpoint**

Eliana Rulli^1^, Francesca Ghilotti^1,2^, Elena Biagioli^1^, Luca Porcu^1^, Mirko Marabese^3^, Maurizio D’Incalci^4^, Rino Bellocco^2,5^, Valter Torri^1^

**Supplementary information**

**Figure S1:** Flowchart of studies selection

**Figure S2:** Trials in which the proportional hazard assumption is violated (1): Log-log plot (on the left side) – Forest plot (on the right side)

**Figure S3:** Trials in which the proportional hazard assumption is violated (2): Log-log plot (on the left side) – Forest plot (on the right side)

**Figure S4:** Trials in which the proportional hazard assumption is violated (3): Log-log plot (on the left side) – Forest plot (on the right side)

**Figure S5:** Hazard ratio versus restricted mean survival time (RMST) ratio; RMST ratio is calculated as ratio between RMST in the control arm and RMST in the experimental arm; the linear regressions between HR and RMST ratio according to PH testing are represented by the dashed lines and the respective equations.

**Table S1:** Procedure used to identify eligible studies

**Table S2:** Characteristics of articles included in the analysis

**Table S3**: Characteristics of articles included in the analysis (2)

Table S1: Procedure used to identify eligible studies

| **Database** | **Query**  **no.** | **Search terms** |
| --- | --- | --- |
| PubMed | 1 | "Carcinoma, Non-Small-Cell Lung/drug therapy"[Mesh] |
|  | 2 | NSCLC[Title/Abstract] |
|  | 3 | (((tumour*[Title/Abstract] OR tumor*[Title/Abstract] OR cancer*[Title/Abstract] OR carcinoma*[Title/Abstract] OR neoplasm*[Title/Abstract]) AND "Non small cell"[Title/Abstract]) AND Lung[Title/Abstract]) |
|  | 4 | ((("VEGF inhibitor"[Title/Abstract] OR "Vascular Endothelial Growth Factor Receptor inhibitor"[Title/Abstract] OR "tyrosine kinase inhibitor"[Title/Abstract] OR erlotinib[Title/Abstract] OR gefitinib[Title/Abstract] OR iressa[Title/Abstract] OR afatinib[Title/Abstract] OR icotinib[Title/Abstract] OR bevacizumab[Title/Abstract] OR avastin[Title/Abstract] OR vandetanib[Title/Abstract] OR endostar[Title/Abstract] OR endostatin[Title/Abstract] OR rh-endostatin[Title/Abstract] OR rhendostatin[Title/Abstract] OR sorafenib[Title/Abstract] OR sunitinib[Title/Abstract] OR cediranib[Title/Abstract] OR nintedanib[Title/Abstract] OR pazopanib[Title/Abstract] OR axitinib[Title/Abstract] OR crizotinib[Title/Abstract])) OR (chemotherapy[Title/Abstract] OR vinorelbine[Title/Abstract] OR navelbine[Title/Abstract] OR cisplatin[Title/Abstract] OR gemcitabine[Title/Abstract] OR paclitaxel[Title/Abstract] OR carboplatin[Title/Abstract] OR docetaxel[Title/Abstract] OR pemetrexed[Title/Abstract] OR alimta[Title/Abstract] OR methotrexate[Title/Abstract] OR abraxane[Title/Abstract])) |
|  | 5 | ((#1) OR #2) OR #3 |
|  | 6 | (#5) AND advanced |
|  | 7 | ((((RCT[Title/Abstract] OR "Randomized clinical trial"[Title/Abstract] OR "Randomized controlled trial"[Title/Abstract] OR "Randomised clinical trial"[Title/Abstract] OR "Randomised controlled trial"[Title/Abstract])) OR "Randomized Controlled Trial"[Publication Type])) |
|  | 8 | ((#4 AND #6 AND #7)) |
|  | 9 | (#8) Filters: Publication date from 2004/01/01 |
| Embase | 1 | 'non small cell lung cancer'/exp/dm_dt |
|  | 2 | lung:ab,ti |
|  | 3 | 'non small cell':ab,ti |
|  | 4 | tumour:ab,ti OR tumor:ab,ti OR cancer:ab,ti OR carcinoma:ab,ti OR neoplasm:ab,ti OR tumours:ab,ti OR tumors:ab,ti OR cancers:ab,ti OR carcinomas:ab,ti OR neoplasms:ab,ti |
|  | 5 | nsclc:ab,ti |
|  | 6 | #2 AND #3 AND #4 |
|  | 7 | #1 OR #5 OR #6 |
|  | 8 | 'vegf':ab,ti OR 'vascular endothelial growth factor receptor':ab,ti OR 'vascular endothelial growth factor receptors':ab,ti OR 'tyrosine kinase':ab,ti OR erlotinib:ab,ti OR gefitinib:ab,ti OR iressa:ab,ti OR afatinib:ab,ti OR icotinib:ab,ti OR bevacizumab:ab,ti OR avastin:ab,ti OR vandetanib:ab,ti OR endostar:ab,ti OR endostatin:ab,ti OR 'rh endostatin':ab,ti OR rhendostatin:ab,ti OR sorafenib:ab,ti OR sunitinib:ab,ti OR cediranib:ab,ti OR nintedanib:ab,ti OR pazopanib:ab,ti OR axitinib:ab,ti OR crizotinib:ab,ti OR chemotherapy:ab,ti OR vinorelbine:ab,ti OR navelbine:ab,ti OR cisplatin:ab,ti OR gemcitabine:ab,ti OR paclitaxel:ab,ti OR carboplatin:ab,ti OR docetaxel:ab,ti OR pemetrexed:ab,ti OR alimta:ab,ti OR methotrexate:ab,ti OR abraxane:ab,ti |
|  | 9 | #7 AND #8 |
|  | 10 | #9 AND advanced |
|  | 11 | rct OR 'randomized clinical trial' OR 'randomised clinical trial' OR 'randomized controlled trial' OR 'randomised controlled trial' OR 'randomized controlled trial'/de |
|  | 12 | #10 AND #11 |
|  | 13 | # 12 AND [2004-2015]/py |
|  | 14 | #13 AND [embase]/lim NOT [medline]/lim |

**Table S2:** Characteristics of articles included in the analysis

| **Author** | **Year** | **Arms** | **Control**  **arm** | **Sper. arm 1** | **Sper. arm 2** | **Control**  **arm**  **Code** | **Sper.**  **arm 1**  **Code** | **Sper.**  **arm 2**  **Code** | **Control**  **arm**  **treatment**  **schedule** | **Sper.**  **Arm 1**  **Treatment**  **schedule** | **Sper.**  **Arm 2**  **Treatment**  **schedule** | **Treatment**  **line** |
| --- | --- | --- | --- | --- | --- | --- | --- | --- | --- | --- | --- | --- |
| Ardizzoni | 2012 | 2 | pem | pem > car |  | 1 | 1 |  | 4 cycles | 4 cycles |  | II |
| Barlesi | 2013 | 2 | bev | bev+pem |  | 2 | 1+2 |  | prog | prog |  | maint |
| Belani | 2014 | 3 | pem+cis | pem+cis+axi(daily) | pem+cis+  axi(2-19) | 1 | 1+3 | 1+3 | 6 cycles | 6 cycles > axi to prog | 6 cycles > axi to prog | II |
| Chouaid | 2011 | 2 | pem | pem+zibo |  | 1 | 1+3 |  | na | na |  | II |
| de Boer | 2011 | 2 | pem | pem+van |  | 1 | 1+3 |  | 6 cycles | 6 cycles > van to prog |  | II |
| Dittrich | 2014 | 2 | pem | pem+erl |  | 1 | 1+3 |  | na | prog |  | II |
| Edelman | 2013 | 2 | car+pac | car+ixa |  | 1 | 1 |  | 6 cycles | 6 cycles |  | I |
| Flotten | 2012 | 2 | vin+car | vin+gem |  | 1 | 1 |  | 3 cycles | 3 cycles |  | I |
| Garassino | 2013 | 2 | doc | erl |  | 1 | 3 |  | prog | prog |  | II |
| Garon | 2014 | 2 | doc | doc+ram |  | 1 | 1+2 |  | prog | prog |  | II |
| Gridelli | 2007 | 2 | pem | sequential  pem/gem |  | 1 | 1 |  | 8 cycles | 8 cycles |  | I |
| Gridelli | 2014 | 2 | gem | gem+van |  | 1 | 1+3 |  | 6 cycles or prog | 6 cycles or prog |  | I |
| Groen | 2013 | 2 | erl | erl+sun |  | 3 | 3 |  | 18 cycles or prog | 18 cycles or prog |  | II |
| Hanna | 2004 | 2 | doc | pem |  | 1 | 1 |  | prog | prog |  | II |
| Heigener | 2014 | 2 | car+vin | erl |  | 1 | 3 |  | 6 cycles | prog |  | I |
| Herbst | 2011 | 2 | erl | erl+bev |  | 3 | 2+3 |  | prog | prog |  | II |
| Herbst | 2010 | 2 | doc | doc+van |  | 1 | 1+3 |  | prog | prog |  | II |
| Janne | 2013 | 2 | doc | doc+sel |  | 1 | 1+3 |  | 6 cycles | 6 cycles > sel to prog |  | II |
| Johnson | 2013 | 2 | bev | bev+erl |  | 2 | 2+3 |  | prog | prog |  | maint |
| Karampeazis | 2013 | 2 | erl | pem |  | 3 | 1 |  | prog | 6 cycles |  | II/III |
| Kawaguchi | 2014 | 2 | doc | erl |  | 1 | 3 |  | prog | prog |  | II/III |
| Kelly | 2012 | 2 | erl | pra |  | 3 | 1 |  | prog | prog |  | II/III |
| Kim | 2013 | 2 | doc | doc+cet |  | 1 | 1+2 |  | 6 cycles or prog | 6 cycles or prog > cet to prog |  | II/III |
| Kim | 2008 | 2 | doc | gef |  | 1 | 3 |  | prog | prog |  | II/III |
| Kubota | 2008 | 2 | pac+car | vin+gem>doc |  | 1 | 1 |  | 6 cycles | 6 cycles |  | I |
| Lee | 2010 | 2 | doc | gef |  | 1 | 3 |  | 6 cycles or prog | prog |  | II |
| Li | 2014 | 2 | pem+car | pem+car>gef |  | 1 | 1+3 |  | 4 cycles | 4 cycles > gef for 6 months |  | I |
| Lilenbaum | 2005 | 2 | pac | pac+car |  | 1 | 1 |  | 6 cycles cycles | 6 cycles |  | I |
| Maruyama | 2008 | 2 | doc | gef |  | 1 | 3 |  | prog | prog |  | II |
| Mok | 2009 | 2 | pac+car | gef |  | 1 | 3 |  | 6 cycles or prog | 6 cycles or prog |  | I |
| Natale | 2011 | 2 | erl | van |  | 3 | 3 |  | prog | prog |  | II/III |
| Niho | 2012 | 2 | car+pac | car+pac+bev |  | 1 | 1+2 |  | 6 cycles or prog | prog |  | I |
| Okamoto | 2010 | 2 | car+pac | car+S-1 |  | 1 | 1 |  | 6 cycles or prog | 6 cycles or prog |  | I |
| Paccagnella | 2006 | 2 | pac+car | pac+car+gem |  | 1 | 1 |  | 6 cycles or prog | 6 cycles or prog |  | I |
| Patel | 2013 | 2 | induction:  bev+car+pac;  maint:  bev | induction:  bev+car+pem;  maint:  bev+pem |  | 2 | 1+2 |  | prog | prog |  | I |
| Paz-Ares | 2012 | 2 | cis+gem;  maint:  placebo | cis+gem+sorafenib;  maint:  sorafenib |  | 1 | 1+3 |  | 6 cycles | 6 cycles > sorafenib prog |  | I |
| Paz-Ares | 2013 | 3 | car+pac | car+pac+  con(15mg/Kg) | car+pac+  con(3mg/Kg) | 1 | 1+2 | 1+2 | 6 cycles or prog | 6 cycles or prog > con prog | 6 cycles or prog > con prog | I |
| Pirker | 2009 | 2 | cis+vin | cis+vin+cet |  | 1 | 1+2 |  | 6 cycles | 6 cycles > cet to prog |  | I |
| Quoix | 2011 | 2 | vin  OR  gem | car+pac |  | 1 | 1 |  | 5 cycles | 4 cycles |  | I |
| Ramalingam | 2014 | 2 | erl | dac |  | 3 | 3 |  | prog | prog |  | II/III |
| Ramlau | 2012 | 2 | doc | doc+afi |  | 1 | 1+3 |  | prog | prog |  | II |
| Reck | 2009 | 3 | cis+gem | cis+gem+  bev(7.5mg) | cis+gem+  bev(15mg) | 1 | 1+2 | 1+2 | 6 cycles or prog | 6 cycles or prog | 6 or prog | I |
| Reck | 2013 | 2 | car+pac | car+pac+tig |  | 1 | 1+2 |  | 6 cycles | 6 cycles > tig |  | I |
| Reck | 2014 | 2 | doc | doc+nin |  | 1 | 1+3 |  | prog | prog |  | II |
| Rosell | 2012 | 2 | cis+doc  OR  cis+gem | erl |  | 1 | 3 |  | 4 cycles or prog | prog |  | I |
| Rudd | 2005 | 2 | mit+ifo+cis | gem+car |  | 1 | 1 |  | 4 cycles | 4 cycles |  | I |
| Scagliotti | 2012 | 2 | pac+car | pac+car+mot |  | 1 | 1+3 |  | 6 cycles or prog | 6 cycles or prog |  | I |
| Scagliotti | 2012 | 2 | erl | erl+sun |  | 3 | 3 |  | prog | prog |  | II/III |
| Seto | 2014 | 2 | erl | erl+bev |  | 3 | 2+3 |  | prog | prog |  | I |
| Shaw | 2013 | 2 | pem  OR  doc | cri |  | 1 | 3 |  | prog | prog |  | II |
| Shi | 2013 | 2 | gef | ico |  | 3 | 3 |  | prog | prog |  | II/III |
| Smit | 2009 | 2 | pem | pem+car |  | 1 | 1 |  | 4 cycles | 4 cycles |  | II |
| Solomon | 2014 | 2 | pem+cis  OR  pem+car | cri |  | 1 | 3 |  | 6 cycles or prog | 6 cycles or prog |  | I |
| Spigel | 2013 | 2 | erl | erl+ona |  | 3 | 2+3 |  | prog | prog |  | II/III |
| Treat | 2010 | 3 | pac+car | gem+car | gem+pac | 1 | 1 | 1 | 6 cycles or prog | 6 cycles or prog | 6 or prog | I |
| Wu | 2014 | 2 | gem+cis | afa |  | 1 | 3 |  | 6 cycles or prog | prog |  | I |
| Wu | 2013 | 2 | gem+pla | gem+pla+erl |  | 1 | 1+3 |  | 6 cycles or prog | 6 cycles or prog > erl |  | I |
| Zukin | 2013 | 2 | pem | car+pem |  | 1 | 1 |  | 4 cycles | 4 cycles |  | I |

Arm code: 1, conventional therapy (drugs causing DNA damage or inhibition of DNA synthesis); 2, biologics (antibodies against growth and angiogenic factors); 3, tyrosine-kinase inhibitor

Abbreviations: +, in addition; >, followed by; afa, afatinib; afl, aflibercept; axi, axitinib; bev, bevacizumab; car, carboplatin; cet, cetuximab; cis, cisplatin; con, conatumumab; cri, crizotinib; dac, dacomitinib; doc, docetaxel; erl, erlotinib; gef, gefitinib; gem, gemcitabine; ico, icotinib; ifo, ifosfamide; ixa, ixabepilone; maint, maintenance; mit, mitomycin; mot, motesanib; na, not available; nin, nintedanib; ona, onartuzumab; pac, paclitaxel; pem, pemetrexed; pra, pralatrexate; prog, until progression; ram, ramucirumab; sel, selumetinib; sor, sorafenib; sper, sperimental; sun, sunitinib; tig, tigatuzumab; van, vandetanib; vin, vinorelbine; zib, zibotentan;

**Table S3**: Characteristics of articles included in the analysis (2)

| Author | Year | Phase | Primary endpoint | Comparison | Number of patients | Journal | Multicentric | Cancer type | Result |
| --- | --- | --- | --- | --- | --- | --- | --- | --- | --- |
| Ardizzoni | 2012 | II | PFS | superiority | 239 | Journal of Clinical Oncology | yes | all | negative |
| Barlesi | 2013 | III | PFS | superiority | 253 | Journal of Clinical Oncology | yes | nonsquamous | positive |
| Belani | 2014 | II | PFS | superiority | 170 | BMC Cancer | yes | nonsquamous | negative |
| Chouaid | 2011 | II | OS | superiority | 66 | Cancer Chemother Pharmacol | yes | nonsquamous | negative |
| De Boer | 2011 | III | PFS | superiority | 534 | Journal of Clinical Oncology | yes | all | negative |
| Dittrich | 2014 | II | PFS | superiority | 159 | European Journal of Cancer | yes | nonsquamous | positive |
| Edelman | 2013 | II | PFS | superiority | 197 | Journal of Clinical Oncology | yes | all | negative |
| Flotten | 2012 | III | OS | superiority | 437 | British Journal of Cancer | yes | all | negative |
| Garassino | 2013 | III | OS | superiority | 219 | Lancet Oncology | yes | EGFR wt | positive |
| Garon | 2014 | III | OS | superiority | 1253 | Lancet | yes | all | positive |
| Gridelli | 2007 | II | TTP | superiority | 87 | Journal of Thoracic Oncology | yes | all | unclear |
| Gridelli | 2014 | II | PFS | superiority | 124 | Journal of Thoracic Oncology | yes | all | positive |
| Groen | 2013 | II | PFS | superiority | 132 | Annals of Oncology | yes | all | negative |
| Hanna | 2004 | III | OS | non inferiority | 571 | Journal of Clinical Oncology | yes | all | equivalent |
| Heigener | 2014 | II | PFS | non inferiority | 238 | Lung Cancer | yes | all | inferiore |
| Herbst | 2011 | III | OS | superiority | 636 | Lancet | yes | all | negative |
| Herbst | 2010 | III | PFS | superiority | 1391 | Lancet Oncology | yes | all | positive |
| Janne | 2013 | II | OS | superiority | 83 | Lancet Oncology | yes | KRAS mutated | negative |
| Johnson | 2013 | III | PFS | superiority | 743 | Journal of Clinical Oncology | yes | all | positive |
| Karampeazis | 2013 | III | TTP | superiority | 332 | Cancer | yes | nonsquamous | negative |
| Kawaguchi | 2014 | III | PFS | superiority | 301 | Journal of Clinical Oncology | yes | all | negative |
| Kelly | 2012 | II | OS | superiority | 201 | Journal of Clinical Oncology | yes | 100 or more cigarettes smoked | negative |
| Kim | 2013 | III | PFS | superiority | 605 | Lancet Oncology | yes | all | negative |
| Kim | 2008 | III | OS | non inferiority in overall population (superiority in patients with high EGFR gene copy number) | 1433 | Lancet | yes | all | equivalent.  negative for patients with high EGFR gene copy number |
| Kubota | 2008 | III | OS | superiority | 393 | Lancet Oncology | yes | all | negative |
| Lee | 2010 | III | PFS | superiority | 161 | Clinical Cancer Research | yes | all | positive |
| Li | 2014 | II | PFS | superiority | 60 | Annals of Surgical Oncology | no | EGFR mutated IIIA-N2 NSCLC | positive |
| Lilenbaum | 2005 | III | OS | superiority | 561 | Journal of Clinical Oncology | yes | all | negative |
| Maruyama | 2008 | III | OS | non inferiority | 489 | Journal of Clinical Oncology | yes | all | negative |
| Mok | 2009 | III | PFS | non inferiority | 1217 | The New England Journal of Medicine | yes | adenocarcinoma, non smokers | positive |
| Natale | 2011 | III | PFS | superiority | 1240 | Journal of Clinical Oncology | yes | all | negative |
| Niho | 2012 | II | PFS | superiority | 175 | Lung Cancer | yes | nonsquamous | positive |
| Okamoto | 2010 | III | OS | non inferiority | 563 | Journal of Clinical Oncology | yes | all | equivalent |
| Paccagnella | 2006 | II/III | OS | superiority | 324 | Journal of Clinical Oncology | yes | all | positive |
| Patel | 2013 | III | OS | superiority | 939 | Journal of Clinical Oncology | yes | nonsquamous | negative |
| Paz-Ares | 2012 | III | OS | superiority | 772 | Journal of Clinical Oncology | yes | nonsquamous | negative |
| Paz-Ares | 2013 | II | PFS | superiority | 172 | Journal of Thoracic Oncology | yes | all | negative |
| Pirker | 2009 | III | OS | superiority | 1125 | Lancet | yes | EGFR-expressing | positive |
| Quoix | 2011 | III | OS | superiority | 451 | Lancet | yes | all | positive |
| Ramalingam | 2014 | III | PFS | superiority | 878 (519) | Lancet Oncology | yes | all (KRAS wt) | negative |
| Ramlau | 2012 | III | OS | superiority | 913 | Journal of Clinical Oncology | yes | nonsquamous | negative |
| Reck | 2009 | III | PFS | superiority | 1043 | Journal of Clinical Oncology | yes | nonsquamous | positive |
| Reck | 2013 | II | PFS | superiority | 97 | Lung Cancer | yes | all | negative |
| Reck | 2014 | III | PFS | superiority | 1314 | Lancet Oncology | yes | all | positive |
| Rosell | 2012 | III | PFS | superiority | 173 | Lancet Oncology | yes | EGFR mutated | positive |
| Rudd | 2005 | III | OS | superiority | 422 | Journal of Clinical Oncology | yes | all | positive |
| Scagliotti | 2012 | III | OS | superiority | 1090 (890) | Journal of Clinical Oncology | yes | nonsquamous (adenocarcinoma) | negative |
| Scagliotti | 2012 | III | OS | superiority | 960 | Journal of Clinical Oncology | yes | all | negative |
| Seto | 2014 | II | PFS | superiority | 152 | Lancet Oncology | yes | nonsquamous EGFR mutated | positive |
| Shaw | 2013 | III | PFS | superiority | 347 | The New England Journal of Medicine | yes | ALK-positive | positive |
| Shi | 2013 | III | PFS | non inferiority | 395 | Lancet Oncology | yes | all | equivalent |
| Smit | 2009 | II | PFS | superiority | 240 | Journal of Clinical Oncology | yes | all | positive |
| Solomon | 2014 | III | PFS | superiority | 343 | The New England Journal of Medicine | yes | ALK-positive | positive |
| Spigel | 2013 | II | PFS | superiority | 137 (66) | Journal of Clinical Oncology | yes | all (MET-positive) | positive |
| Treat | 2010 | III | OS | superiority | 1135 | Annals of Oncology | yes | all | negative |
| Wu | 2014 | III | PFS | superiority | 364 | Lancet Oncology | yes | adenocarcinoma EGFR mutated | positive |
| Wu | 2013 | II | PFS | superiority | 451 | Lancet Oncology | yes | all | positive |
| Zukin | 2013 | III | OS | superiority | 205 | Journal of Clinical Oncology | yes | all | positive |
