# Supplementary figures and images for: Assessment of proportional hazard assumption in aggregate data: a systematic review on statistical methodology in clinical trials using time-to-event endpoint
Source: Br J Cancer. 2018 Nov 13;119(12):1456–63. doi: 10.1038/s41416-018-0302-8 (PMC6288087; doi:10.1038/s41416-018-0302-8)

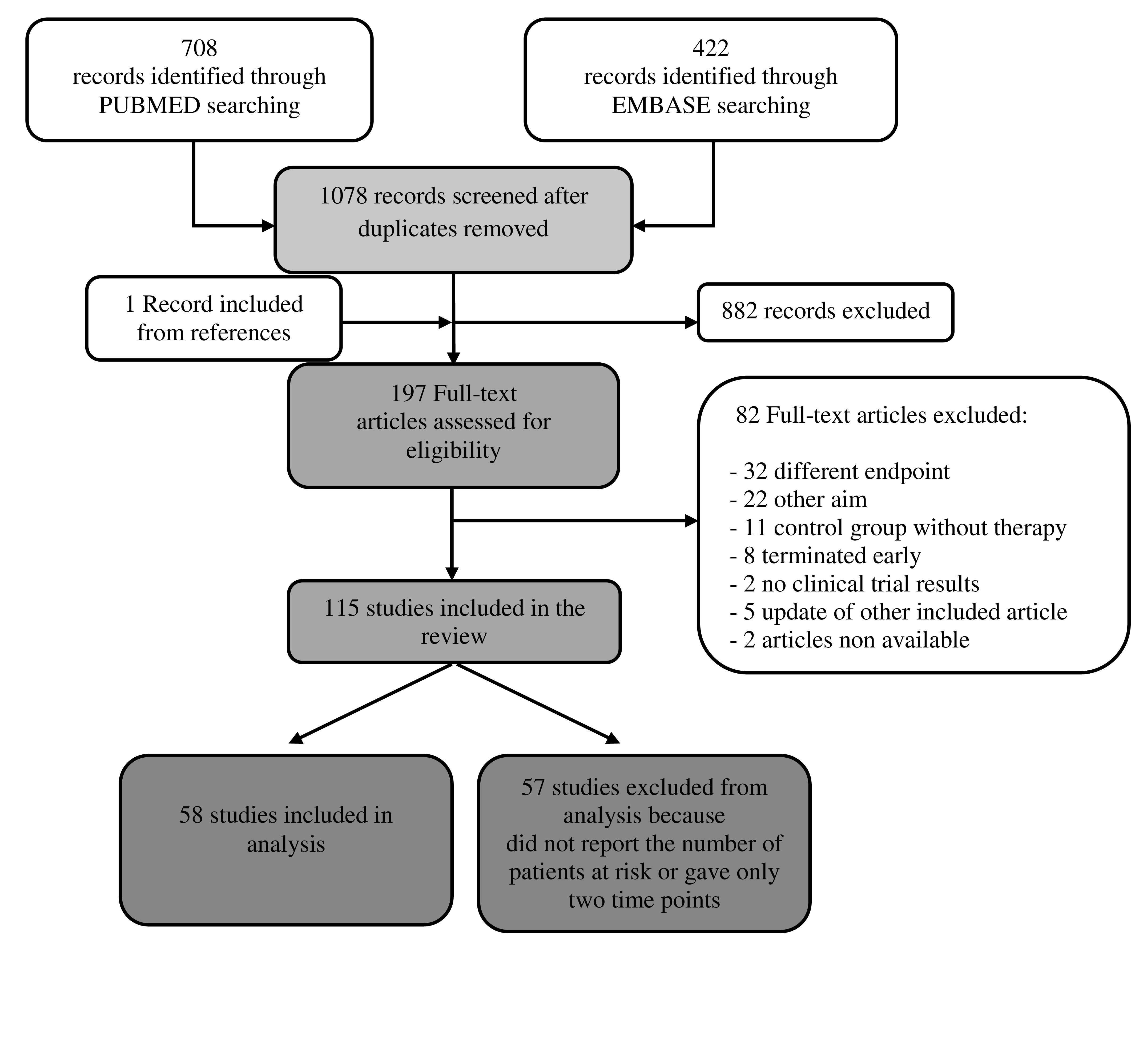

Supplement: Supplementary file 2 — supplementary figure S1 [file 41416_2018_302_MOESM2_ESM.tif]

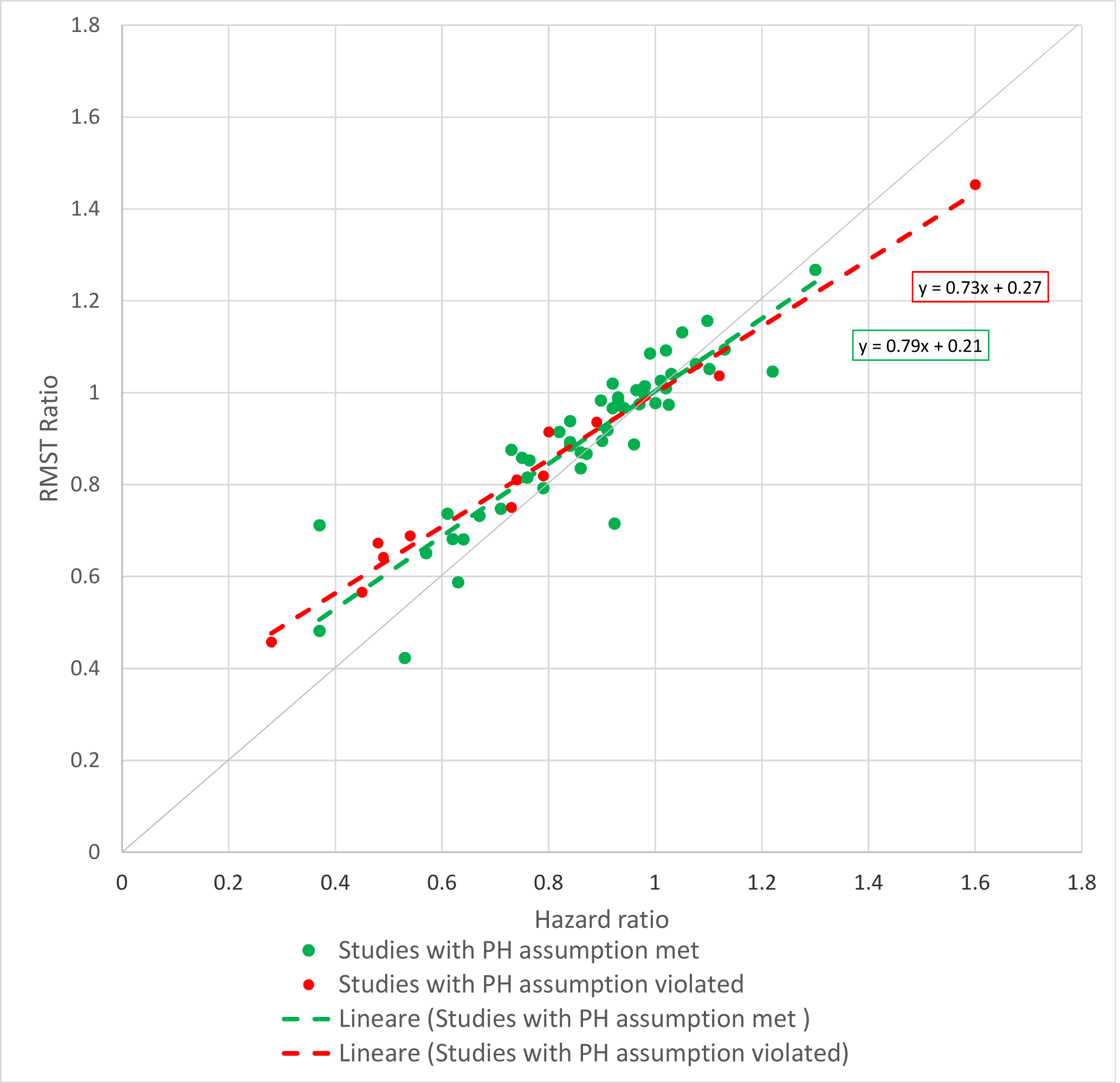

Supplement: Supplementary file 6 — supplementary figure S5 [file 41416_2018_302_MOESM6_ESM.tif]
