# Supplementary material for: Assessment of proportional hazard assumption in aggregate data: a systematic review on statistical methodology in clinical trials using time-to-event endpoint
Source: Br J Cancer. 2018 Nov 13;119(12):1456–63. doi: 10.1038/s41416-018-0302-8 (PMC6288087; doi:10.1038/s41416-018-0302-8)
Supplement: Supplementary file 7 — permission to pubblish fig 1d [file 41416_2018_302_MOESM7_ESM.pdf]

AMERICAN SOCIETY OF CLINICAL ONCOLOGY LICENSE TERMS AND CONDITIONS

Sep 18, 2018

This Agreement between Mirko Marabese ("You") and American Society of Clinical Oncology ("American Society of Clinical Oncology") consists of your license details and the terms and conditions provided by American Society of Clinical Oncology and Copyright Clearance Center.

License Number	4431791149783
License date	Sep 18, 2018
Licensed Content Publisher	American Society of Clinical Oncology
Licensed Content Publication	Journal of Clinical Oncology
Licensed Content Title	ATLAS: Randomized, Double-Blind, Placebo-Controlled, Phase IIIB Trial Comparing Bevacizumab Therapy With or Without Erlotinib, After Completion of Chemotherapy, With Bevacizumab for First-Line Treatment of Advanced Non–Small-Cell Lung Cancer
Licensed Content Author	Bruce E. Johnson, Fairooz Kabbinavar, Louis Fehrenbacher, et al
Licensed Content Date	Nov 1, 2013
Licensed Content Volume	31
Licensed Content Issue	31
Type of Use	Journal/Magazine/Newsletter
Requestor type	Author (orig article)
Format	Print and electronic
Portion	Figure/table
Number of figures/tables	1
Geographic Rights	Worldwide
Will you be translating?	No
Number of copies	5000
Title	Assessment of proportional hazard assumption in aggregate data: a systematic review on statistical methodology in clinical trials using time-to-event endpoint
Author	E. Rulli, F. Ghilotti, E. Biagioli, L. Porcu, M. Marabese, M. D’Incalci, R. Bellocco, V. Torri
Publication	British Journal of Cancer
Publisher	Springer Nature
Expected publication date	Nov 2018
Expected size	5
Portions	Figure 3
Requestor Location	Mirko Marabese<br>via La Masa, 19<br><br>Milan, mi 20156<br>Italy<br>Attn: Mirko Marabese
Billing Type	Invoice
Billing Address	Mirko Marabese<br>via La Masa, 19<br><br>Milan, Italy 20156<br>Attn: Mirko Marabese
Total	**0.00 EUR**
Terms and Conditions	
