# Supplementary material for: Assessment of proportional hazard assumption in aggregate data: a systematic review on statistical methodology in clinical trials using time-to-event endpoint
Source: Br J Cancer. 2018 Nov 13;119(12):1456–63. doi: 10.1038/s41416-018-0302-8 (PMC6288087; doi:10.1038/s41416-018-0302-8)
Supplement: Supplementary file 8 — permission to pubblish fig 1a [file 41416_2018_302_MOESM8_ESM.pdf]

AMERICAN SOCIETY OF CLINICAL ONCOLOGY ORDER DETAILS

Sep 18, 2018

Order Number	501425086
Order date	Aug 31, 2018
Licensed Content Publisher	American Society of Clinical Oncology
Licensed Content Publication	Journal of Clinical Oncology
Licensed Content Title	Randomized Phase III Trial of Maintenance Bevacizumab With or Without Pemetrexed After First-Line Induction With Bevacizumab, Cisplatin, and Pemetrexed in Advanced Nonsquamous Non–Small-Cell Lung Cancer: AVAPERL (MO22089)
Licensed Content Author	Fabrice Barlesi, Arnaud Scherpereel, Achim Rittmeyer, et al
Licensed Content Date	Aug 20, 2013
Licensed Content Volume	31
Licensed Content Issue	24
Type of use	Journal/Magazine/Newsletter
Requestor type	Author (orig article)
Format	Print and electronic
Portion	Figure/table
Number of figures/tables	1
Geographic Rights	Worldwide
Will you be translating?	No
Number of copies	5000
Title	Assessment of proportional hazard assumption in aggregate data: a systematic review on statistical methodology in clinical trials using time-to-event endpoint
Author	E. Rulli, F. Ghilotti, E. Biagioli, L. Porcu, M. Marabese, M. D'Incalci, R. Bellocco, V. Torri
Publication	British Journal of Cancer
Publisher	Springer Nature
Expected publication date	Nov 2018
Expected size	5
Portions	Figure 1A
Requestor Location	Mirko Marabese<br>via La Masa, 19<br><br>Milan, mi 20156<br>Italy<br>Attn: Mirko Marabese
Total	**0.00 EUR**
Terms and Conditions	

General Terms& Conditions

Permission is granted upon the requester's compliance with the following terms and conditions:

1. A credit line will be prominently placed in your product(s) and will include: for books-the author, book title, editor, copyright holder, year of publication; for journals-the author, title of article, title of journal, volume number, issue number, and the inclusive pages. The credit line must include the following wording: “Reprinted with permission. © (Year of publication being used) American Society of Clinical Oncology. All rights reserved.” The citation format must be
